# Delayed Impairment of Postural, Physical, and Muscular Functions Following Downhill Compared to Level Walking in Older People

**DOI:** 10.3389/fphys.2020.544559

**Published:** 2020-10-21

**Authors:** Mathew William Hill, Edyah-Ariella Hosseini, Abbie McLellan, Michael James Price, Stephen Ronald Lord, Anthony David Kay

**Affiliations:** ^1^Centre for Sport, Exercise and Life Sciences, Coventry University, Coventry, United Kingdom; ^2^Falls, Balance and Injury Research Centre, Neuroscience Research Australia, University of New South Wales, Sydney, NSW, Australia; ^3^Centre for Physical Activity and Life Sciences, University of Northampton, Northampton, United Kingdom

**Keywords:** fatigue, muscle damage, falls, balance, aging, functional performance, walking

## Abstract

Transient symptoms of muscle damage emanating from unaccustomed eccentric exercise can adversely affect muscle function and potentially increase the risk of falling for several days. Therefore, the aims of the present study were to investigate the shorter- and longer-lasting temporal characteristics of muscle fatigue and damage induced by level (i.e., concentrically biased contractions) or downhill (i.e., eccentrically biased contractions) walking on postural, physical, and muscular functions in older people. Nineteen participants were matched in pairs for sex, age and self-selected walking speed and allocated to a level (*n* = 10, age = 72.3 ± 2.9 years) or downhill (*n* = 9, age = 72.1 ± 2.2 years) walking group. Postural sway, muscle torque and power, physical function (5× and 60 s sit-to-stand; STS), and mobility (Timed-Up-and-Go; TUG) were evaluated at baseline (pre-exercise), 1 min, 15 min, 30 min, 24 h, and 48 h after 30 min of level (0% gradient) or downhill (−10% gradient) walking on a treadmill. Following downhill walking, postural sway (+66 to 256%), TUG (+29%), 60 s STS (+29%), five times STS (−25%) and concentric power (−33%) did not change at 1–30 min post exercise, but were significantly different (*p* < 0.05) at 24 and48 h post-exercise when compared to baseline (*p* < 0.05). Muscle torque decreased immediately after downhill walking and remained impaired at 48 h post-exercise (−27 to −38%). Immediately following level walking there was an increase in postural sway (+52 to +98%), slower TUG performance (+29%), fewer STS cycles in 60 s (−23%), slower time to reach five STS cycles (+20%) and impaired muscle torque (−23%) and power (−19%) which returned to baseline 30-min after exercise cessation (*p* > 0.05). These findings have established for the first time distinct impairment profiles between concentric and eccentric exercise. Muscle damage emanating from eccentrically biased exercise can lead to muscle weakness, postural instability and impaired physical function persisting for several days, possibly endangering older adult’s safety during activities of daily living by increasing the risk of falls.

## Introduction

Falls among older people represents a substantial health and social priority. Falling often leads to progressive functional decline, the development of comorbidities and the start of dependency (Okubo et al., 2017). It is well established that ∼35% of adults aged 65 years and over fall annually (Stalenhoef et al., 2002). Although the etiology of falling is complex (Rubenstein, 2006), several modifiable risk factors have been documented including diminished balance and mobility (Delbaere et al., 2010), a progressive reduction in muscle strength and/or power (Orr et al., 2006) and poor cognitive function (Mirelman et al., 2012). Exercise can protect against a loss of physical (Cadore et al., 2013; Chou et al., 2012; Giné-Garriga et al., 2014) and cognitive function (Colcombe and Kramer, 2003) in older age. However, an inevitable consequence of being physically active is short-term muscle fatigue (Vuillerme et al., 2002). Exercise may acutely increase the risk of falling by negatively influencing postural, muscular, and physical functions (Helbostad et al., 2010). However, the importance of such transient muscle fatigue as a fall-risk factor in older people is not well understood.

Fatigue (or tiredness) is a common complaint among older adults with more than 50% of people over 70 years reporting fatigue during daily activities (Avlund, 2010). One area that has garnered attention recently is the effects of daily activities (such as walking) on intrinsic fall risk factors (i.e., balance, strength, mobility, and cognitive function) (Sturnieks et al., 2018). Although walking is an essential prerequisite to quality of life and independent living (Lee and Buchner, 2008) most falls occur during ambulation (Berg et al., 1997; Robinovitch et al., 2013; Talbot et al., 2005). Consequently, careful consideration of activities that may influence gait are important. During uphill or level walking the lower extremity muscles predominantly perform concentric contractions, resulting in a high metabolic cost (Malm et al., 2004). Consequently, walking can affect several factors associated with falls including an increase in postural sway (Hill et al., 2015; Donath et al., 2013, 2015; Walsh et al., 2018), altered gait characteristics (Nagano et al., 2014), slower reaction time (Morrison et al., 2016) and the decreased muscle strength (Morrison et al., 2016), and power (Foulis et al., 2017). Concentric-dominant exercise, such as level walking, provokes a high energetic metabolism and an intracellular accumulation of metabolic by-products (Green, 1997). These metabolites can adversely influence sensorimotor coupling mechanisms responsible for postural control (Paillard, 2012). However, fatigue-related functional impairments are often transient with performance measures returning to baseline within 20 min (Hill et al., 2015; Donath et al., 2013, 2015). Therefore the relative impact of short-term fatigue induced by level or uphill walking may be limited.

Daily activities are not isolated to concentric contractions but can also incorporate eccentric contractions (such as walking downhill or descending flights of stairs). Conversely to concentric exercise, these activities can result in non-metabolic fatigue. For example, downhill walking elicits a considerably lower oxygen uptake than level or uphill walking when matched at the same speed (Laursen et al., 2000). However, this exercise imposes greater loading on the muscle-tendon complex (Howatson et al., 2011) during braking to control the rate of knee flexion (Maeo et al., 2017). One of the hallmarks of eccentric muscle contractions is the short-term manifestation of myocellular damage and disruption to extrafusal and intramuscular fibers (Raastad et al., 2010). Muscle damage often results in a concomitant reduction in muscle force (Proske and Allen, 2005) and proprioception (Paschalis et al., 2008). These changes typically present ∼6 h after exercise and peak at one to three days thereafter (Paschalis et al., 2008). The amalgamation of muscle damaging effects lasting for several days is likely to be problematic for postural and physical functions and may substantially increase the risk of sustaining a fall for a prolonged period. Consequently, it is likely the magnitude of change and recovery of muscular, physical function and postural profiles associated with prolonged downhill walking may differ from those observed following level walking.

To date, no study has compared the effects of concentric- versus eccentric-dominant exercise-induced fatigue on fall risk factors among older adults; information likely valuable for elucidating some of the fundamental aspects of task-dependent muscle fatigue on fall risk factors. Therefore, the aims of the present study were to investigate the shorter- (up to 30 min) and longer-lasting (up to 48 h) temporal characteristics of muscle fatigue induced by level (i.e., concentric contractions) and downhill (i.e., eccentric contractions) walking on physical, muscular and postural functions associated with fall risk in older people. We hypothesized that level walking, characterized by metabolic fatigue, would provoke an immediate increase in postural sway, a reduction in physical functional performance and reduced isometric strength and concentric power, recovering to baseline levels within 30 min of exercise cessation. Secondly, we hypothesized that downhill walking, characterized by non-metabolic fatigue (i.e., muscle damage), would elicit a delayed (24–48 h) recovery of postural sway, a reduction physical functional performance and impaired muscle function.

## Materials and Methods

### Participants

Effect sizes (Cohen’s *d*) were calculated from similar studies from mean changes in the anteroposterior centre of pressure (COP) displacement (*d* = 2.08), mediolateral COP displacement (*d* = 1.79) (Hill et al., 2015) and knee extensor concentric power (*d* = 1.47) (Foulis et al., 2017). Sample size was estimated using an *a priori* power analysis (G^∗^ Power software [Version 3.1.9.4]) for knee extensor concentric power (i.e., variable with the smallest effect size to avoid bias) (statistical power = 0.80, alpha = 0.05, effect size = 1.47) and revealed that a total of nine participants in each group would be sufficient to detect significant effects of level and downhill walking on measures of postural, muscular, and physical functions (Faul et al., 2009). A total of 19 community-dwelling older adults (Table 1) were recruited with no prior experience of eccentric training and able to walk without the use of an assistive device. All participants had some previous experience of walking on a motorized treadmill but none had any experience of walking downhill on a treadmill. Participants were excluded if they were unable to stand unassisted or had a history of neurological (e.g., stroke, Parkinson’s disease), musculoskeletal (e.g., tendinitis), cognitive impairment (e.g., dementia) and/or cardiovascular or pulmonary diseases (e.g., coronary heart disease, chronic obstructive pulmonary disease). A screening medical questionnaire revealed that no participants had any conditions that would preclude them from participating. Following ethical approval by Coventry University’s Ethical Review Board (Ref; P90521) and prior to any data collection, all participants gave written and informed consent. All risks associated with the experimental procedures were explained before testing began with the study undertaken in accordance with the Declaration of Helsinki (1964).

**TABLE 1:** Mean ± SD sample characteristics.

Sample characteristics	Level walking (*n* = 10)	Downhill walking (*n* = 9)
Sex (male/female)	(5/5)	(4/5)
Age (years)	72.3 ± 2.9	72.1 ± 2.2
Body mass (kg)	72.8 ± 13.7	72.2 ± 9.0
Height (m)	1.65 ± 0.09	1.64 ± 0.09
BMI (kg⋅m^2^)	26.9 ± 5.0	26.9 ± 4.5
Cognitive status (MMSE)	28.7 ± 1.2	28.4 ± 1.0
Physical activity (hr⋅w^–1^)	5.9 ± 3.5	5.9 ± 3.3
Self-reported fatigue (BFI)	8.5 ± 4.5	8.4 ± 2.5
Falls efficacy (FES-I)	17.4 ± 2.1	17.7 ± 1.0
Self-selected walking speed (km⋅h^–1^)	3.9 ± 1.0	4.0 ± 0.5

*BMI, body mass index; MSSE, Mini Mental State Examination; BFI, Brief Fatigue Inventory; FES-I, Falls Efficacy Scale International.*

### Questionnaires

During the consenting visit, all participants completed questionnaires for self-reported physical activity, concern about falls, fatigue and cognitive function. Participants were moderately active (Table 1), as confirmed by the screening medical and physical activity questionnaire. The 16 item Falls Efficacy Scale (FES-I) measures concern about falling during physical and social activities with each item scored on a 4-point Likert scale [1 = not at all concerned to 4 = very concerned; Yardley et al. (2005)] with cumulative scores of 16–19, 20–27, and 28–64 indicating low, moderate and high concern about falling, respectively (Delbaere et al., 2010). Participants also completed the Mini Mental State Examination (MMSE), consisting of 11 questions to determine cognitive function (Crum et al., 1993). An MMSE score of <24 separates individuals with mild dementia from participants with normal cognitive function (Folstein et al., 1983). Self-reported fatigue was measured using the nine item Brief Fatigue Inventory (BFI) to assess the impact of fatigue on daily functioning (Mendoza et al., 1999). This inventory has been validated in healthy people over 65 years of age (Shuman-Paretsky et al., 2014). Participants rated their fatigue on an 11-point scale (0 = no fatigue, 10 = “as bad as you can imagine”) with higher scores on the BFI corresponding to greater self-reported levels of fatigue.

### Experimental Design

The study was conducted as a repeated-measures between group design with criterion measurements evaluated at baseline (pre-exercise), immediately post-exercise (1-min), and again at 15-min, 30-min, 24 h, and 48 h following exercise interventions. We opted against a within-subject crossover design as the protective effect against muscle damage from a single bout of eccentric exercise (McHugh, 2003) may influence level walking characteristics. Following the consenting visit, all participants completed a familiarization session prior to the experimental trials at least 72 h, but not more than 7 days before the first experimental trial. Anthropometric characteristics, postural sway, muscle function (torque and power), physical function (60 s sit-to-stand; STS) and mobility (timed up and go) were collected during this time (described later). To habituate participants to walking on the treadmill and to ascertain self-selected walking speed for subsequent experimental trials, each participant walked for 5–10 min on the treadmill at a 0% gradient (Roberts et al., 2018). Participants were specifically instructed to walk at a preferred comfortable pace that they felt they could maintain for ∼30 min. The starting speed was 2.5 km⋅h^–1^ with an increase of 0.2 km⋅h^–1^ every 30 s until the participant indicated that the next increment in speed would be too fast. Familiarizing the participants during downhill walking was deliberately avoided as a small volume of non-damaging downhill walking (5 min at −28%) can prevent muscle damage during subsequent prolonged (40 min at −28%) downhill walking (Maeo et al., 2017). All participants were blind to their self-selected walking speed, and the principal investigator adjusted speed in 0.2 km⋅h^–1^ increments in response to instructions from the participant to go “slower” or “faster” with the principal investigator standing next to the treadmill to assist the participants to complete the tests safely (Roberts et al., 2018). Following baseline assessments, participants were allocated to a level (0%) or downhill (−10%) walking intervention. Participants were matched in pairs for sex, age, physical activity levels, and self-selected walking speed to minimize potential confounding factors between groups. Independent-sample *t*-tests revealed no significant difference between groups for any participant characteristic at baseline (*p* > 0.05; Table 1). During the experimental period, participants were instructed not to perform any unfamiliar activities and to avoid any interventions that might influence recovery, such as massage, application of ice packs, or nutritional supplements.

### Posturography

Postural sway was measured during quiet standing as a measure of static balance performance. Each participant performed quiet stance trials while standing on a force platform (AMTI, AccuGait, Watertown, MA, United States) for 30 s. Data were sampled at 100 Hz (AMTI, Netforce) and the total displacement of COP in the anteroposterior and mediolateral directions (both cm) and mean COP velocity (cm⋅s^–1^) were subsequently calculated (AMTI, BioAnalysis, Version 2.2). The validity and reliability of these parameters have previously been established for this sampling duration (Pinsault and Vuillerme, 2009). To ensure continuity during trials, unshod foot position was standardized by instructing participants to stand with the feet together (i.e., Romberg stance). To avoid unnatural postural sway, internal focus of attention and restriction of exploratory behavior, participants were not specifically asked to stand as still as possible. Participants’ arms were left to hang freely by their sides and were instructed to look straight ahead at a target 1.5 m away; adjusted to the eye level of each individual. Throughout all tests, the investigator stayed close to the participants to prevent falling but without interfering with balance performance. Before the walking exercise intervention, participants performed three 30 s trials for each condition of eyes open (EO) and eyes closed (EC), in a counterbalanced order, with the mean of the three trials for each condition used in the subsequent analysis. To capture the immediate effects of fatigue on postural sway, a single EO and EC trial was collected at 1, 15-, and 30-min post-exercise, with longer-term effects measured 24 and 48 h after the exercise bouts with all tests undertaken at the same time of the day (±1 h).

### Mobility and Physical Function

The Timed Up and Go Test (TUG) was used as described by Podsiadlo and Richardson (1991). Participants were instructed to stand up from a chair without using their hands, walk 3 m at a normal pace to a line on the floor, walk back to the chair and sit down. Before performing the TUG, an experienced researcher provided verbal and visual instructions regarding the test procedures. Participants were asked to perform the TUG at their self-selected walking speed. The time taken to complete the test was recorded using a stopwatch (nearest 0.01 s) with a total of three trials recorded with 30 s rest between trials; the fastest trial was included in the subsequent analyses. Two minutes later, the participants completed the 60 s STS test (Strassmann et al., 2013). In addition to the total number of repetitions performed in the 60 s STS, the time taken for the first five repetitions were also recorded. Participants were instructed to sit down on a chair (seat height 45 cm) with arms folded across the chest and feet shoulder width apart. Participants were instructed to stand up fully with complete knee and hip extension and sit down as many times as possible within 60 s, with participants verbally encouraged throughout the duration of the test. Given the onerous nature of the test, only a single trial was recorded before exercise, and at 1, 15-, and 30-min, 24 and 48 h post-exercise. Among community dwelling older people, test-retest reliability [intraclass correlation coefficient (ICC)] of the TUG (ICC = 0.99; Podsiadlo and Richardson, 1991), 60 s STS (ICC = 0.80; Ritchie et al., 2005) and five times STS (ICC = 0.89; Lord et al., 2002) are excellent.

### Muscle Torque and Power

The pre-intervention functional balance and mobility tests were also used as the warm-up for subsequent maximal muscle function tests. All maximal voluntary contractions (MVCs) of the knee extensors were performed on the dominant leg (self-selected by participants) using the Cybex Norm isokinetic dynamometer (Computerized Sports Medicine Inc., United States). Participants were seated comfortably in an upright position with the backrest angle at 100° and the knee flexed to 90°. The lateral femoral epicondyle (i.e., axis of rotation) of the dominant knee was aligned with the axis of rotation of the dynamometer with extraneous movements during MVC’s prevented with restraining straps placed over the trunk, pelvis, thigh and ankle. The knee angle was placed at 90° for all isometric MVC’s (torque; Nm) and initial position for concentric contractions (power; W) with gravity correction performed for all tests. The range of motion for dynamic contractions was 70° (range 90°–160° extension). For familiarization of isokinetic and isometric contractions, participants performed three submaximal (∼70% MVC) isometric and concentric contractions, with 3 min of rest between each contraction type. For all time points, each participant first completed three knee extensor isometric MVCs with each isometric contraction lasting ∼4 s. Following the isometric MVCs, participants completed three concentric MVC’s at 180° s^–1^, with the angular velocity based on their tolerance and ability documented during prior pilot testing. High velocity contractions were chosen because they are functionally relevant to physical performance tasks, such as rising from a chair (Hortobagyi et al., 2003) and changes in high velocity muscle power (270° s^–1^) are associated with increases in chair rise time following level walking in older adults (Foulis et al., 2017).

Participants were instructed to contract maximally over the complete range of motion and rested for 1 min between each set. During each testing session, participants were given verbal encouragement to help to ensure a maximal effort with visual feedback of the torque trace provided. During the three repetitions for both contraction modes, the trial with the highest torque was used in subsequent analysis for each mode.

### Exercise Interventions

**FIGURE 1:**
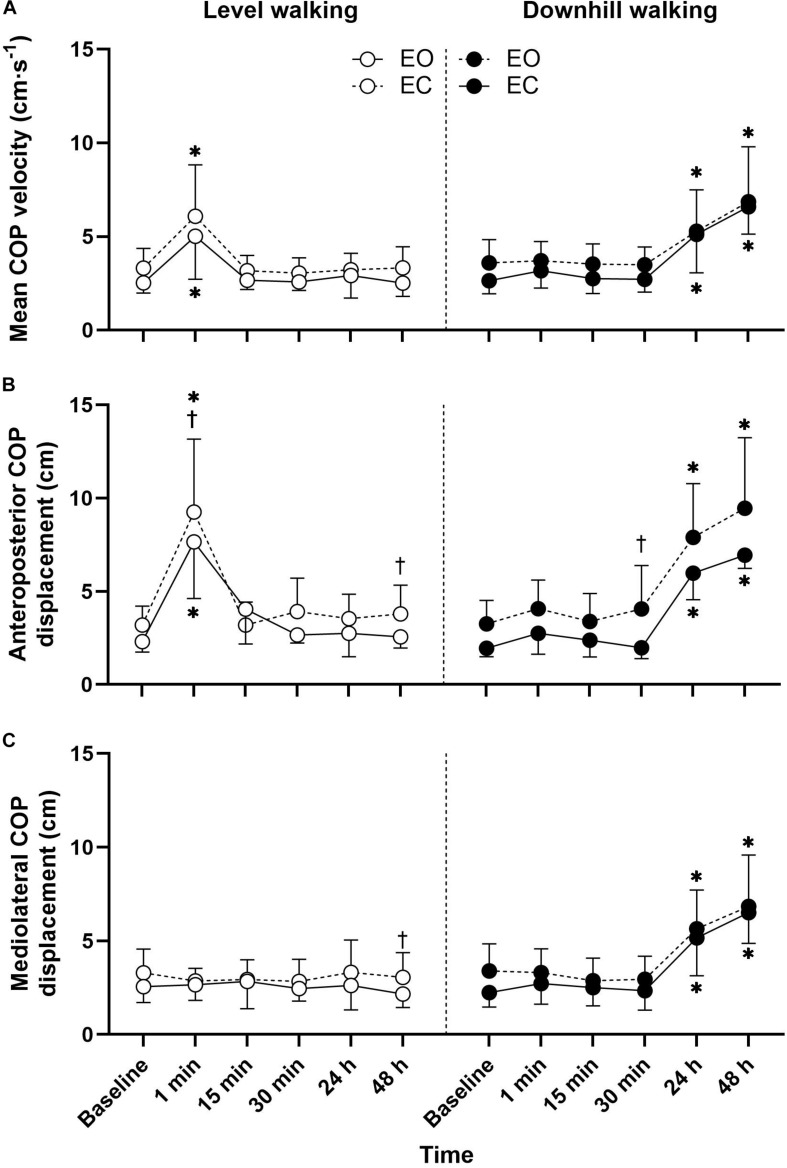
Mean ± SD mean COP velocity **(A)**, anteroposterior COP displacement **(B)**, and mediolateral COP displacement **(C)** responses to 30 min level and downhill walking. ^∗^Significantly different to baseline. ^†^Significant vision effect.

Immediately following baseline assessments, participants completed 30 min of level (0% gradient) or downhill (-10% gradient) walking on an instrumented treadmill (h/p/Cosmos Mercury 4.0; h/p/cosmos Sports & Medical gmbh, Nussdorf-Traunstein, Germany). The −10% gradient was adopted because this is the point at which minimum total body energy costs occurs for downhill walking (Wanta et al., 1993). Participants wore a safety harness attached to an automated overhead suspension arm during the interventions to prevent possible falls from the treadmill (Figure 1). Starting with a treadmill speed of 1.5 km⋅h^–1^, the velocity was progressively increased until participants reached their pre-determined (during familiarization) self-selected walking speed after ∼30–60 s. As with the baseline assessment, all participants were blind to their self-selected walking speed, with the principal investigator adjusting speed in 0.2 km⋅h^–1^ increments and standing next to the treadmill to assist the participants to complete the tests safely. Participants wore their own comfortable sports shoes and clothes, however compression garments were not permitted. During both level and downhill walking, participants were instructed to walk at their most comfortable stride length and stride rate and not to change it throughout the trial (Maeo et al., 2017). The 30 min walking time was selected as previous studies have reported that this is sufficient to elicit muscle fatigue-related effects on postural control (Hill et al., 2015; Foulis et al., 2017; Walsh et al., 2018). Additionally, this exercise duration aligns with the American College of Sports Medicine recommendations for health benefits in older people (Nelson et al., 2007).

### Physiological and Perceptual Responses

During the walking test, expired gases were analyzed using a breath-by-breath online analysis system (MetaMax, Cortex Biophsik, Borsdorf, Germany) for oxygen uptake ($\dot{V}$O_2_), pulmonary ventilation ($\dot{V}$_E_) and respiratory exchange ratio (RER). Expired gas data were subsequently calculated and averaged over the final 20 s of each 5 min interval. Oxygen and carbon dioxide sensors and the gas turbine were calibrated prior to each test according to the manufacturer’s guidelines. Heart rate (HR) was continually monitored (Polar Electro, Oy, Finland) and recorded in the final 10 s of each 5 min interval. The 15-point (6–20) Borg scale was used to determine cardiorespiratory (heart and lungs; RPE_C_) and local (working muscles; RPE_L_) ratings of perceived exertion (Borg, 1982). RPE_L_ and RPE_C_ were obtained at the same time as HR.

### Statistical Analysis

Data were analyzed using SPSS version 25.0 (IBM Inc., Chicago, IL, United States). All measures are reported as mean ± SD. Separate two-way mixed-model ANOVAs examined the effects of time (×6; pre-exercise, 1, 15, 30-min, 24, and 48 h post-exercise) and group (×2; level vs. downhill walking) on TUG, STS, muscle torque, and power. Three-way ANOVA were used for postural sway metrics (group × time × vision). Separate two-way mixed-model ANOVAs were also used to examine the effects of time (×7; pre-exercise, 5, 10, 15, 20, 25, 30-min post-exercise) and group (×2; level vs. downhill walking) on cardiorespiratory and perceptual variables. For all measures, normality (Shapiro–Wilk test) and homogeneity of variance (Levene’s test) and sphericity (Macuhley’s test) were confirmed prior to undertaking parametric tests. Where significant differences were detected, *post hoc* analyses with Bonferroni-adjusted α for multiple comparisons were conducted to determine the location of any significant differences. Effect sizes are reported as partial eta-squared value (η_p_^2^) for ANOVA and as Cohen’s d (*d*) for pairwise comparisons, with 0.2, 0.6, 1.2, and 2.0 indicating small, medium, large and very large effects, respectively (Hopkins et al., 2009). Pearson’s product moment correlation coefficients (*r*) were computed to quantify the relationship between the absolute change score data in all variables. Statistical significance for all tests was accepted at *p* < 0.05.

### Reliability

Within-session reliability was examined using ICC and coefficients of variation (CV) during baseline conditions between the second and third trials. No significant differences (p > 0.05) were detected between any measure of postural sway, TUG, isometric strength or concentric power. Moderate-to-high ICC and low-to-moderate CV were calculated for anteroposterior COP displacement (EO; ICC = 0.82, CV = 11.1%, EC; ICC = 0.92, CV = 10.9%), mediolateral COP displacement (EO; ICC = 0.95, CV = 8.1%, EC; ICC = 0.94, CV = 9.5%), mean COP velocity (EO; ICC = 0.99, CV = 3.1%, EC; ICC = 0.97, CV = 5.0%), TUG (ICC = 0.99, CV = 1.5%), isometric strength (ICC = 0.97, CV = 4.3) and concentric power (ICC = 0.99, CV = 5.8%). Given that participants completed only one STS test before exercise, ICC’s and CV’s are not reported for the five times STS or 60 s STS tests.

## Results

### Postural Sway

Postural sway responses are illustrated in Figure 1. The three-way ANOVA revealed significant group × time interactions for all postural sway outcomes (Table 2). Follow up *post hoc* analysis indicated that the anteroposterior COP displacement (*p* < 0.001, *d* = 2.45) and mean COP velocity (*p* < 0.001, *d* = 1.49) with eyes open were significantly greater 1-min after level walking compared to pre-exercise, returning to pre-exercise levels within 15 min (*p* > 0.05). During the eyes closed condition the anteroposterior COP displacement (*p* < 0.001, *d* = 2.12) and mean COP velocity (*p* = 0.002, *d* = 1.33) were significantly greater 1-min following level walking compared to pre-exercise, returning to pre-exercise levels within 15 min (*p* > 0.05).

**TABLE 2:** Group × time × vision repeated measures ANOVA of postural sway responses to level and downhill walking.

	Anteroposterior COP displacement			Mediolateral COP displacement			Mean COP velocity		
	*F*	*p*	η_p_^2^	*F*	*p*	η_p_^2^	*F*	*p*	η_p_^2^
Group	0.831	0.363	0.004	21.832	0.001	0.097	13.882	0.001	0.064
Time	21.718	0.001	0.349	12.969	0.001	0.242	13.663	0.001	0.252
Vision	21.709	0.001	0.097	6.551	0.011	0.031	10.044	0.002	0.047
Group × Time	29.517	0.001	0.421	11.224	0.001	0.217	16.866	0.001	0.293
Group × Vision	0.842	0.360	0.004	0.875	0.351	0.004	0.211	0.647	0.001
Time × Vision	0.938	0.457	0.023	0.155	0.978	0.004	0.225	0.951	0.006
Group × Time × Vision	0.491	0.783	0.012	0.959	0.444	0.023	0.159	0.977	0.004

Following downhill walking, *post hoc* analysis indicated that with the eyes open the mediolateral COP displacement (*p* < 0.001, *d* = 1.90), anteroposterior COP displacement (*p* < 0.001, *d* = 3.83), and mean COP velocity (*p* < 0.001, *d* = 1.61) increased at 24 h post-exercise when compared to baseline and did not recover to baseline levels after 48-h recovery (all *p* < 0.001). For the eyes closed condition, the mediolateral COP displacement (*p* = 0.031, *d* = 1.26), anteroposterior COP displacement (*p* < 0.001, *d* = 2.08) increased at 24 h post-exercise when compared to baseline values, did not recover to baseline levels after 48 h recovery (all *p* < 0.001).

### Physical Function

**FIGURE 2:**
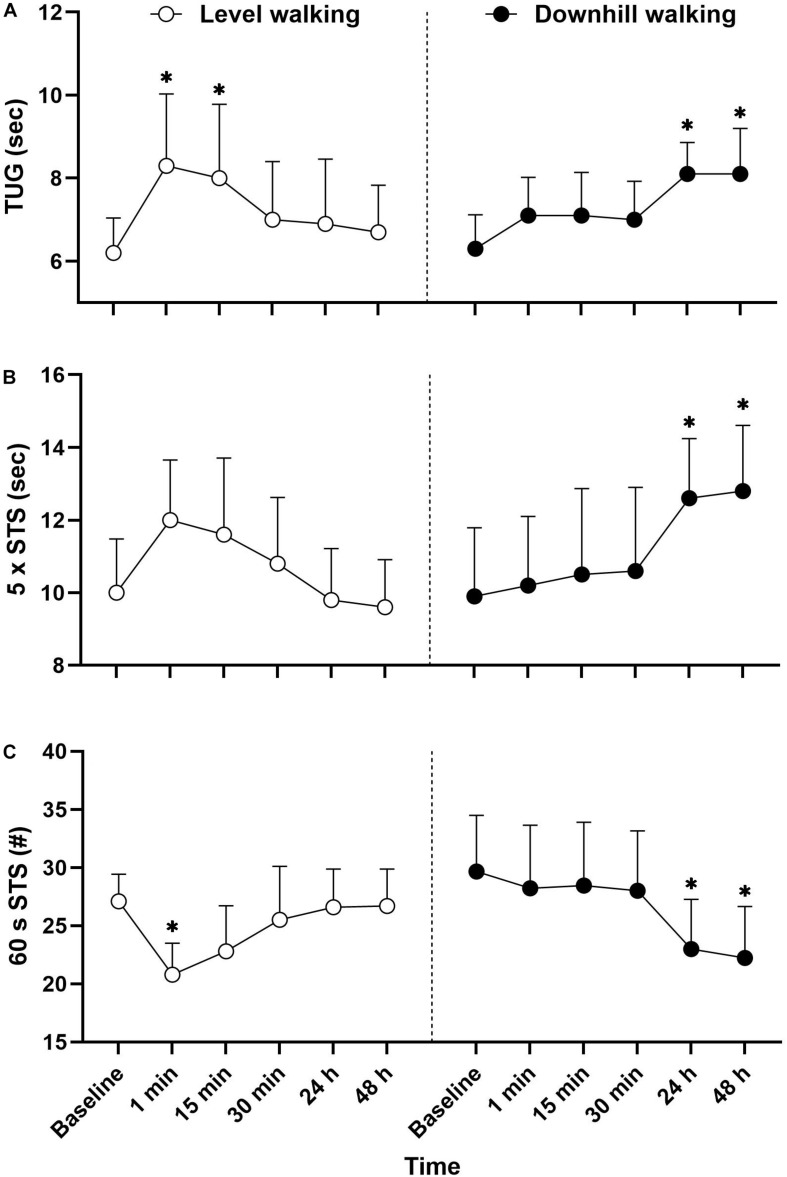
Mean ± SD TUG **(A)**, 5 times STS **(B)**, and 60 s STS **(C)** responses to 30 min level and downhill walking. ^∗^Significantly different to baseline.

There were statistically significant interactions for 60 s STS (*F*_(__5_,_102__)_ = 6.209, *p* < 0.001, η_p_^2^ = 0.233), five times STS (*F*_(__5_,_102__)_ = 5.992, *p* < 0.001, η_p_^2^ = 0.227) and TUG (*F*_(__5_,_102__)_ = 3.318, *p* = 0.008, η_p_^2^ = 0.140) (Figure 2). Immediately following level walking there was a reduction in the number of STS in 60 s (p = 0.017, *d* = 2.50) and TUG increased (p = 0.003, *d* = 1.56), with the TUG remaining significantly slower 15 min post-exercise (p = 0.019, *d* = 1.31). Following downhill walking, there was a reduction in the number of STS in 60 s (p = 0.016, *d* = 1.45), whilst the time to reach 5 STS cycles (*p* = 0.042, *d* = 1.45) and TUG (*p* = 0.050, *d* = 0.92) both increased at 24 h post-exercise. The 60 s STS (*p* = 0.004, *d* = 1.54), five times STS (*p* = 0.019, *d* = 1.50) and TUG (*p* = 0.004, *d* = 1.83) remained significantly different to baseline values at 48 h post-exercise.

### Muscle Function

**FIGURE 3:**
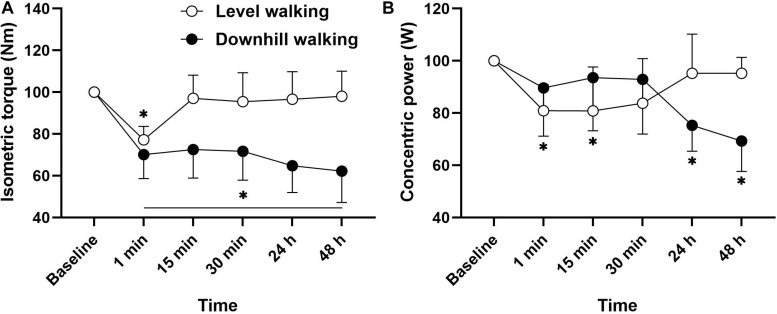
Mean ± SD changes in isometric muscle force **(A)** and concentric power **(B)** from pre-exercise value (100%) before (baseline), during a short term recovery (1–30 min) and up to 48 h after 30 min of level and downhill walking.

The analysis revealed a statistically significant group × time interaction for isometric MVC (*F*_(__5_,_102__)_ = 7.227, *p* < 0.001, η_p_^2^ = 0.262). Figure 3 follow up *post hoc* analysis revealed that compared to baseline, there was a reduction in isometric MVIC 1 min after level walking (*p* < 0.001, *d* = 1.44), returning to baseline after 15 min recovery (*p* > 0.05). Following downhill walking, there was an immediate reduction in MVIC, which remained significantly different to baseline throughout the recovery period (*p* < 0.001, *d* = 1.50–2.15). A significant time × group interaction was also revealed for concentric power (*F*_(__5_,_102__)_ = 6.608, *p* < 0.001, η_p_^2^ = 0.245). *Post hoc* analyses revealed that compared to baseline, there was a reduction in concentric power at 1 min (*p* = 0.042, *d* = 0.73) and 15 min (*p* = 0.041, *d* = 0.70) following level walking. Following downhill walking, the analysis revealed that compared to baseline, there was a reduction in isometric concentric power at 24 h (*p* = 0.004, *d* = 1.02) and 48 h (*p* < 0.001, *d* = 1.32) post-downhill walking. As part of our initial exploratory analyses we performed correlational analysis to determine the relationships among changes in muscle, physical and postural functions. There were no significant associations between changes in muscle and physical function with any postural sway outcomes (*p* > 0.05, *r* = 0.11–0.30).

### Physiological Responses

**FIGURE 4:**
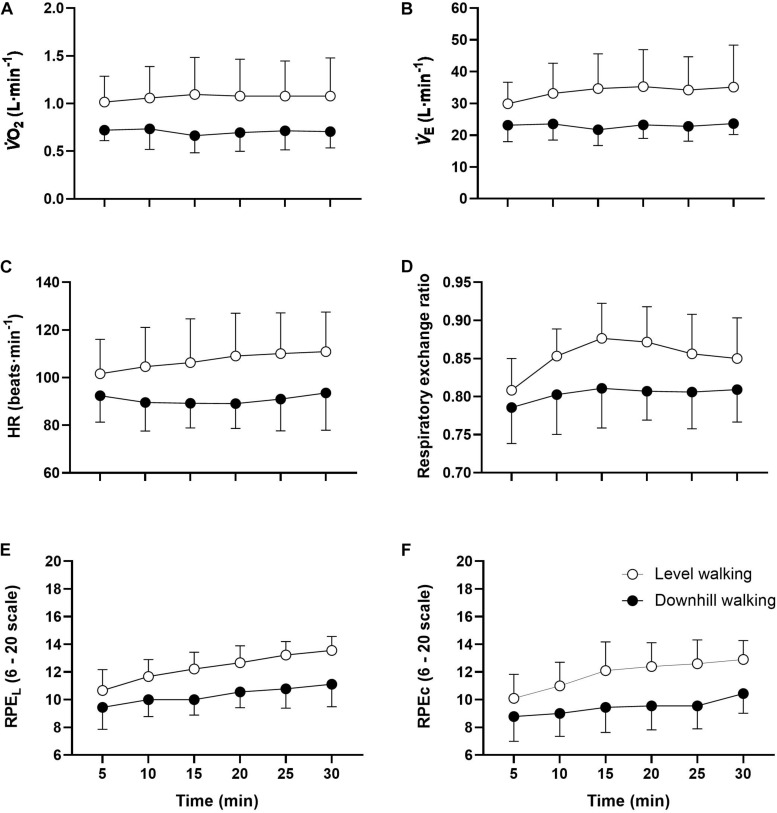
Mean ± SD $\dot{\text{V}}$O_2_ **(A)**, $\dot{\text{V}}$E **(B)**, HR **(C)**, RER **(D)**, RPE_L_ **(E)**, and RPE_C_ **(F)** responses to 30 min level and downhill walking. All responses were greater during level than downhill walking (*p* < 0.001).

Although the analyses revealed no statistically significant group × time interactions for any physiological outcomes (*p* > 0.05), main group effects were observed for all variables (Table 3). Follow up *post hoc* analyses revealed that with the exception of $\dot{\text{V}}$_E_, HR, RER, and RPE_C_ at minute 5, all responses were greater throughout the exercise trials in the level compared to downhill walking group (Figure 4). The analysis also revealed main effects of time for RPE_L_ (*F*_(__5_,_102__)_ = 13.035, *p* < 0.001 η_p_^2^ = 0.291) and RPE_C_ (*F*_(__5_,_102__)_ = 4.202, *p* = 0.002, η_p_^2^ = 0.171). Follow up *post hoc* analyses revealed that compared to 5 min, RPE_L_ was greater at 20 min (*p* = 0.008), 25 min (*p* < 0.001), and 30 min (*p* < 0.001) exercise. Similarly, RPE_C_ was greater at 20 min (*p* = 0.050), 25 min (*p* = 0.022), and 30 min (*p* = 0.006) compared to 5 min.

**TABLE 3:** Repeated measures ANOVA of physiological responses to level and downhill walking.

Analysis	Group × Time ANOVA		
Group × time	*F*	*p*	η_p_^2^
$\dot{\text{V}}$O_2_	0.127	0.986	0.006
$\dot{\text{V}}$_E_	0.336	0.890	0.016
HR	0.324	0.898	0.016
RER	0.467	0.800	0.022
RPE_L_	0.678	0.641	0.032
RPE_C_	0.649	0.663	0.031
**Group**			
$\dot{\text{V}}$O_2_	44.364	<0.001	0.303
$\dot{\text{V}}$_E_	46.768	<0.001	0.314
HR	7537.469	<0.001	0.250
RER	26.302	<0.001	0.205
RPE_L_	75.619	<0.001	0.426
RPE_C_	55.440	<0.001	0.352

## Discussion

This experiment shows that the recovery profiles of postural, physical and muscular functions associated with eccentrically biased exercise differ from those observed following concentrically biased exercise. In accordance with our first hypothesis, the relative impact of short-term fatigue induced by concentrically biased exercise (i.e., metabolic fatigue during level walking) is limited, with a full recovery of postural, physical and muscle functions occurring within 30 min of exercise cessation. In support of our second hypothesis, following eccentrically biased exercise (i.e., muscle damage following downhill walking), not only was the impairment of postural, physical and muscular functions delayed until 24 h post-exercise, these functions remained impaired for at least 48 h post-exercise. The delayed impairment and recovery of postural, physical and muscular functions following low-intensity downhill walking suggest that eccentrically biased contractions could affect daily activities and increase the risk of falling for several days. Collectively, these findings identify distinct temporal profiles which have important practical applications for physical therapists, exercise professionals and geriatricians to provide guidance on behavior following exercise modes likely to differentially elevate fall risk.

### Physiological and Perceptual Responses

It has previously been shown that $\dot{\text{V}}$O_2_ is ∼25% lower during 15 min downhill (–10%) compared to level (0%) walking among older people (Navalta et al., 2004; Gault et al., 2013). In the present study, $\dot{\text{V}}$O_2_ was approximately 34% lower in the downhill compared to the level walking group, accompanied by a lower HR (–32%), $\dot{\text{V}}$_E_ (–15%), and RER (–6%). Additionally, both local and central RPE were consistently two to three points lower during downhill walking (i.e., “very light” to “light”) compared to level walking (i.e., “somewhat hard”). Therefore, we can confirm that the level walking group experienced significantly greater demands on metabolic, cardiovascular and pulmonary systems and elicited a greater perceived exertion compared to the downhill walking group. The underlying physiological process rendering downhill walking less metabolically demanding than level walking involves differences in the energetics of the cross-bridge cycles and the elastically stored energy is released from the muscle-tendon complex (Nishikawa, 2016).

### Fatigue and Recovery Profiles Following Eccentric Exercise

To our knowledge, this is the first study to attempt to identify differences in the effects of concentric- versus eccentrically biased exercise on postural, physical and muscular functions in older adults. Contrary to the level walking group (discussed later), the downhill walking group presented with symptoms of muscle damage. Specifically, maximal isometric muscle force decreased by 35% at 24 h after downhill walking and remained lower (−38%) than the baseline at 48 h post-exercise. Similarly, compared to baseline, concentric power decreased by 25 and 31% at 24 and 48 h, respectively. Although these changes are typical following downhill walking (> 40 min) in healthy young adults (Ahmadi et al., 2008; Maeo et al., 2015; Maeo et al., 2017; Nakayama et al., 2019), limited data exist describing changes in muscle force following downhill walking in older people. Gault et al. (2011) observed a 15% decline in maximal isometric voluntary contraction of the knee extensor muscles 48 h following a 30 min downhill (−10%) treadmill walk at a self-selected walking speed (4.6 km⋅hr^–1^). These changes in isometric muscle torque were considerably less than the changes observed in the present study. These observations may suggest that muscle damage was present following the downhill walking protocol performed in the present study.

Changes in postural sway did not present until 24 h after downhill walking and remained altered at 48 h post-exercise; findings that broadly align with studies undertaken in young adults (Twist et al., 2008). Multiple mechanisms may account for the delayed impairment and recovery of postural control following eccentric exercise-induced muscle damage. First, an increase in postural sway following downhill walking may have resulted from reduced isometric muscle force generation capacity. Weaker muscles require the activation of larger motor units to achieve the same force. Crucially, large motor neurons have less fine control (Saxton et al., 1995), requiring a greater recruitment following eccentric exercise to compensate for the reduction in muscle force (Davies and White, 1981). Consequently, eccentric exercise can lead to an increase in physiological tremor for 24 h after eccentric exercise (Saxton et al., 1995), which may explain the delayed increase in postural sway at 24 h in the present study (Kouzaki and Masani, 2012). This problem might also be exacerbated by the fact that eccentrically biased exercise preferentially recruits and damages fast twitch motor units (Brockett et al., 2002), which could affect the ability to react quickly to large amounts of body sway. Secondly, the temporal changes in postural sway observed in the present study are consistent with recovery profiles of neuromuscular impairments (i.e., force and joint position sense) following eccentric exercise reported in previous studies. For example, several authors have reported that muscle spindles and golgi tendinous receptors become desensitized after 24 h following eccentric exercise-induced muscle damage (Brockett et al., 1997; Paschalis et al., 2007; Saxton et al., 1995), with impairments persisting for several days. These findings suggest that metabolite accumulation associated with concentrically biased exercise cannot be attributed to the delayed and long lasting impairments in postural sway reported here. Whilst the deficits in muscle and joint mechanoreceptors following eccentric exercise-induced muscle damage remains unclear (Torres et al., 2010), it is clear that muscle spindles and golgi tendinous receptors contribute to joint position and movement (Brockett et al., 1997). Finally, it is possible the high ground reaction impact forces during downhill ambulation (Gottschall and Kram, 2005) provoke substantial damage to the plantar cutaneous receptors. It is well established that there is a functional relationship between plantar cutaneous afferents and maintenance of upright stance (Meyer et al., 2004). Regardless of the underlying mechanisms, given that increased postural sway has been shown to be predictive of falls (Piirtola and Era, 2006; Johansson et al., 2017), these findings suggest that exercise-induced muscle damage might impair balance control and lead to a long term “window” of increased fall risk.

Downhill walking involves a substantial eccentric component that causes considerable muscle damage (Nottle and Nosaka, 2005) and a subsequent reduction in muscle strength (Ahmadi et al., 2008; Girard et al., 2018) that typically presents ∼6 h after exercise and peaks at one to three days thereafter. On this basis, we hypothesized that downhill walking would elicit a marked reduction in the performance of the TUG and STS, given their relationship with muscle strength (Bohannon et al., 2010; Coelho-Junior et al., 2018). In addition to the concentric contractions required to stand up, the TUG and STS tasks also involve eccentric contractions of the knee extensors to control lowering of the body to the seated position. Importantly, the delayed impairments in TUG and STS performance followed the same profile as the changes in concentric power. This is not surprising given that Hortobagyi et al. (2003) observed a peak knee extension velocity of 138° s^–1^ in older adults performing a chair rise, emphasizing that fast velocity concentric actions are functionally relevant to physical performance tasks. The delayed impairment and recovery of power reported here is most likely explained by damage induced excitation-contraction uncoupling as a result of reduced release of calcium (Power et al., 2010) and damage to contractile fibers resulting in a reduced shortening velocity. These structural disruptions to excitation-contraction coupling are likely responsible for the delayed impairment and recovery of functional performance following eccentric exercise. The reduced ability to perform the STS and TUG for several days after eccentric exercise may lead to functional impairments during daily activities. As already discussed, there is evidence that older people perform many daily activities close to their maximum capability (Bieryla et al., 2009; Hortobagyi et al., 2003). From a practical perspective, many precarious, high risk tasks (e.g., descending stairs or walking downhill), rely on eccentric muscle contractions (LaStayo et al., 2014). Therefore, exercise professionals, therapists and practitioners should be aware of the negative effects of eccentric exercise-induced muscle damage Further studies are required to develop interventions to minimize exercise-induced muscle damage, especially in older people.

### Fatigue and Recovery Profiles Following Concentric Exercise

Our findings are consistent with previous studies that have reported a transient increase in postural sway (anteroposterior COP displacement and mean COP velocity) immediately following level treadmill walking (Hill et al., 2015; Donath et al., 2013, 2015; Foulis et al., 2017; Walsh et al., 2018). The worsening of postural control immediately following treadmill walking, followed by the rapid recovery of performance has been linked to the accumulation of metabolic by-products (i.e., hydrogen ions, inorganic phosphate or adenosine diphosphate; Paillard, 2012). Metabolic-fatigue can provoke a number of disturbances at the peripheral level [e.g., decreased muscular excitability, increased force fluctuation and deceleration of the afferent conduction velocity (Enoka and Duchateau, 2008; Hunter et al., 2004)], which can deteriorate the accuracy of the sensory proprioceptive information and/or decreases muscular system efficiency (Nardone et al., 1997).

The present findings complement previous work by demonstrating a reduction in isometric force and concentric power following 30 min of level waking (Foulis et al., 2017). Here, we found deficits in isometric torque (–23%) and concentric power (–20%) immediately following level walking, returning to baseline after 15 min and 30 min recovery, respectively. Our observed power and strength deficits were greater than those reported by Foulis et al. (2017) (isometric strength; –8% and concentric power [270°⋅s^–1^]; –13%). Whilst we found a similar reduction in muscle strength and power, contrary to the findings of Foulis et al. (2017), we additionally observed a reduction in the number of STS cycles in 60 s and slower TUG performance following level walking, returning to baseline after 15 and 30 min, respectively. During baseline, participants in the present study completed the TUG in 6.2 ± 0.8 s, times at the faster end of the normative spectrum (7.1–9.0 s) for community-dwelling older adults aged 60–69 years (Bohannon, 2006). Therefore, although unlikely to be of clinical relevance to increase the risk of a fall, the 2.1 s increase in the TUG test indicates a that fatigue negatively contributed to one or more of the tasks of standing up, sitting down, walking or turning, indicating a general decline in mobility. Considering that older adults may use up to 80% of their maximal leg strength to rise of a chair (Hortobagyi et al., 2003), it is not surprising that we observed a reduction in performance of the 60 s STS test following level walking. However, we observed no change in the performance of the five times STS test following fatigue, which aligns with previous research (Foulis et al., 2017). Taken together, the relatively short lasting effects (<30 min) of level walking on muscular, postural and physical functions support the notion that there is an acute post-exercise period in which older people are at an increased risk of falls.

### Practical Implications

There has been considerable attention directed toward the potential applications of eccentric exercise in the last decade, mainly due to the substantial improvements in muscle mass and strength than can be achieved (Hody et al., 2019). Crucially, because of the high force- low cost attributes, eccentric exercise may be ideally suited to exercise-intolerant individuals, such as older people (LaStayo et al., 2003). Indeed, it has been demonstrated that eccentric exercise training elicits superior adaptations in muscle strength/power, muscle mass and physical performance (i.e., mobility) when compared with concentric training in this population (LaStayo et al., 2003; Mueller et al., 2009; Gault and Willems, 2013; Kay et al., 2020). However, given the high muscle forces that can be achieved, it is not surprising that this exercise can cause muscle damage. Our findings show that muscle damage resulting from eccentric exercise can lead to muscle weakness, postural instability and impaired physical function which can persist for several days, endangering older adult’s safety during daily activities and potentially increase the risk of falls. Nevertheless, there is now emerging evidence that muscle damage is not an inevitable consequence of eccentric contractions. For example, low intensity eccentric exercise can elicit protective effects on muscle damage markers induced by high intensity eccentric exercise (Maeo et al., 2017).

### Limitations and Future Directions

We acknowledge a number of study limitations. First, whilst we chose our outcomes (i.e., mobility, balance and muscle weakness) for their associations with fall risk, muscle fatigue and/or damage may also affect fall risk by factors that were not ascertained in this study, such as gait disturbances, impaired cognitive function, and poorer recovery from an unexpected trip or slip. Secondly, we failed to observe a full recovery of postural, muscular and physical functions 48 h after eccentric exercise-induced muscle damage. Therefore, the full timescale of the development of, and recovery from, muscle damage on fall risk remains unclear. Third, our sample was relatively healthy and homogenous, which may restrict the generalizability of the study, although the samples homogeneity may have limited the influence of potential confounding factors. Frail or less active older people would likely be more susceptible to eccentric exercise-induced muscle damage, leading to a greater increase in fall risk in these groups. Fourth, we did not objectively quantify muscle soreness [delayed onset of muscle soreness (DOMS)] or knee pain. However, we asked all participants if they experienced soreness in any muscles of the lower body; eight out of nine participants in the downhill walking group reported modest soreness in the plantar flexors and quadriceps. Only 1 of out 10 participants reported muscle soreness following level walking. Finally, we did not measure eccentric muscle power. Prior pilot testing revealed that older participants (not included in the study) experienced significant difficulty with learning the technique of attempting to “slow down” the dynamometer arm as it moved toward them. Although we acknowledge familiarization may have allowed for adequate learning, we were reluctant to familiarize participants to eccentric exercise due to the risk of conferring protective adaptations against potential further damage (McHugh, 2003). In light of this final point, future research should aim to develop interventions (i.e., non-damaging pre-conditioning exercises) that can minimize the effects of eccentric exercise-induced muscle damage on fall risk factors, especially in older people. There is a reasonable theoretical basis for expectation that performing eccentric pre-conditioning exercise will reduce the eccentric induced consequences (i.e., the repeat bout effect) on balance performance and risk of fall related accidents.

## Conclusion

This is the first investigation to examine the short-term and long-lasting effects of level and downhill walking on fall risk factors among older people. We have demonstrated that exercise-induced muscle damage elicits impairments in postural, muscular and physical functions. Notably, these impairments did not present until 24 h post-exercise, and remained for at least 48 h post-exercise. The delayed impairment and incomplete recovery of these fall risk factors following eccentrically biased exercise suggest that this type of exercise may increase fall risk for several days. Collectively, these findings have important practical implications for exercise prescription.

## Data Availability Statement

The raw data supporting the conclusions of this article will be made available by the authors, without undue reservation.

## Ethics Statement

The studies involving human participants were reviewed and approved by Coventry University Ethics Committee. The patients/participants provided their written informed consent to participate in this study.

## Author Contributions

MH conceived and designed research, performed the analyses, and wrote the manuscript. MH, E-AH, and AM conducted the experiments. MH, MP, AK, and SL revised the manuscript. All authors read and approved the final manuscript.

## Conflict of Interest

The authors declare that the research was conducted in the absence of any commercial or financial relationships that could be construed as a potential conflict of interest.
